# Severe hyponatremia in an infant with epidermolysis bullosa: a case report

**DOI:** 10.1186/s13256-022-03601-6

**Published:** 2022-10-07

**Authors:** Soheil Dehghani, Boshra Akbarzadeh Pasha, Amirali Karimi, Azadeh Afshin

**Affiliations:** 1 School of Medicine, Tehran University of Medical Sciences, Tehran, Iran; 2 Prevention of Metabolic Disorders Research Center, Research Institute for Endocrine Sciences, Shahid Beheshti University of Medical Sciences, Tehran, Iran; 3 Pediatric Nephrologist, Pediatric Chronic Kidney Disease Research Center, Tehran University of Medical Sciences, Tehran, Iran

**Keywords:** Case report, Electrolyte imbalance, Epidermolysis bullosa, Hyponatremia, Infant

## Abstract

**Background:**

Epidermolysis bullosa is a rare inherited connective tissue disorder compromising cellular junctions. Blister formation is the first manifestation of epidermolysis bullosa. As cellular adhesion is affected, it can affect many organs. Due to compromised skin integrity, water loss and electrolyte imbalances are prevalent in these patients. However, hypernatremia is the usual observed sodium imbalance rather than hyponatremia.

**Case presentation:**

The patient was a 48-day-old Iranian male infant born near term. He was diagnosed with epidermolysis bullosa at 1 month of age. The patient was brought to the pediatrics center with apnea and respiratory distress, and was intubated and admitted to the pediatric intensive care unit. His symptoms started 4 days before the admission with vomiting and poor feeding, and the patient later developed loss of consciousness. Vital signs revealed a pulse rate of 154 beats per minute, respiratory rate of 70 per minute, a temporal temperature of 36.5 °C, nondetectable blood pressure, and oxygen saturation of 96%. The patient was anuric at presentation and was rehydrated. Physical examination showed bolus eruptions all over the body but not in mucosal membranes. Important laboratory findings were white blood cell count of 41,000/mm^3^ with 68% neutrophils, hemoglobin of 10.8 g/dL, platelet count of 856,000/mm^2^, negative C-reactive protein (CRP), blood sugar of 514 mg/dL, urea of 129 mg/dL, sodium of 98 mg/dL, corrected sodium of 105 mg/dL, potassium of 5.5 mg/dL, serum creatinine of 1.7 mg/dL, and serum procalcitonin of more than 75 ng/mL. Urine analysis revealed many red blood cells. Brain computed tomography demonstrated loss of differentiation between gray and white matter and effacement of cortical sulci suggesting severe cytotoxic edema.

We administered 3% hypertonic saline and corrected the plasma sodium levels, and provided the patient with multiple doses of mannitol as well as antibiotics due to the leukocytosis. Subsequently, after 3 days in pediatric intensive care unit, the symptoms of brain edema resolved, and after 4 days, he was weaned from the ventilator and extubated. Later he was discharged from the pediatric intensive care unit.

**Conclusion:**

This study illustrates the possibility of severe hyponatremia in patients with epidermolysis bullosa to clinicians. Although uncommon, knowledge on such possibilities is vital due to the possible detrimental outcomes for patients.

## Background

Epidermolysis bullosa (EB) is an inherited connective tissue disorder that causes tissue fragility and blister formation spontaneously or by minor trauma [1]. Although blister formation is one of the first presentations of EB, systemic symptoms and involvement of other organs have been seen in EB [2]. EB has been divided into four subtypes based on underlying molecular abnormalities and gene mutations, including EB simplex, junctional EB, dystrophic EB, and Kindler EB [3]. One of the main pathophysiological mechanisms of EB that can affect many organs, especially the skin, is the perturbation of cellular adhesion [1], which is involved in the interface between the upper layer of the cellular dermis and the epidermal basement membrane zone [4, 5]. Due to this high tissue fragility and the impairment of skin integrity, many pathogens can enter the body and present their antigens to the immune system. Hence, the immune system will produce antibodies and inflammatory markers. This inflammatory response may cause a variety of diseases, including a tendency to develop chronic inflammations [6, 7], infections, sepsis, electrolyte imbalances, nonhealing wounds or lesions [8], and multiorgan damage (skin, kidney, colon, and heart) [9, 10]. Although the immune system plays a major role in this state by hyperinflammatory response, also immunocompromised condition and using systemic or topical corticosteroid medicine complicate the treatment of EB [11, 12].

According to studies, the prevalence of EB is estimated to vary slightly from country to country [4, 13], but the national EB registry (NEBR) in the USA has reported it to be 11.1 per million population and 19.6 per million live births [14].

As mentioned above, involvement of various organs is expected in EB. Electrolyte imbalances are one of the most common outcomes due to water loss, and sodium imbalances usually presents as hypernatremia. However, we faced a male infant with junctional EB who presented with hyponatremia. This piece of appropriate knowledge about the incidence of this rare condition would help patients and healthcare managers

## Case report

The patient was a 48-day-old Iranian male infant born near term with gestational age of 37^4/7^ weeks, birth weight of 3400 g, and birth head circumference of 37 cm, from nonconsanguineous parents, by elective cesarean section. His mother had gestational diabetes mellitus, but the patient had no family history of bullous disorders or other dermal disorders. After birth, he was hospitalized at the neonatal intensive care unit (NICU) for 14 days due to respiratory distress syndrome. He also had bullous skin eruptions from birth. Therefore, he underwent skin biopsy at 1 month old and was diagnosed with EB.

**Fig. 1:**
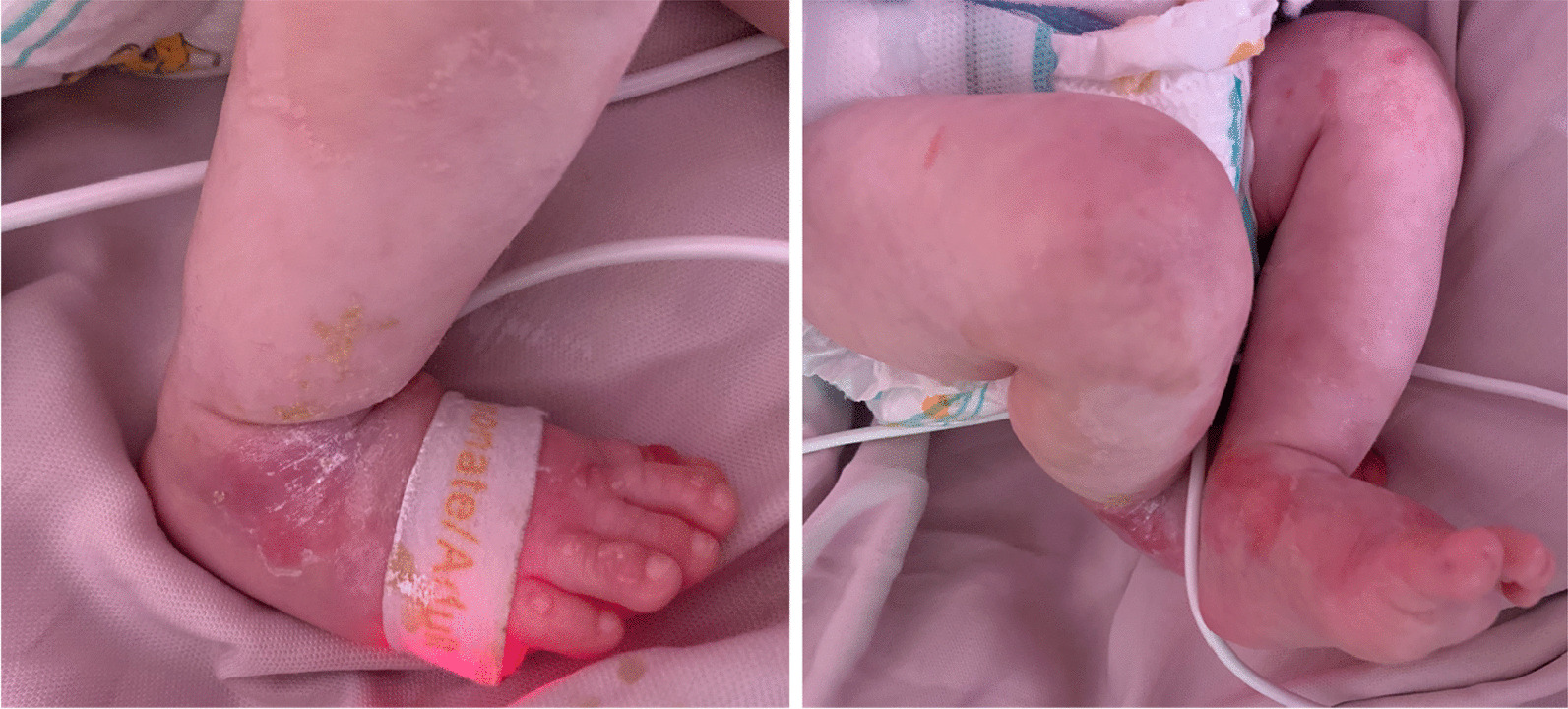
Patient’s feet with erythematous patches and bullous eruptions on dorsal surface of toes

He was brought to the pediatrics center with apnea and respiratory distress, was intubated on the emergency ward, and then admitted to the pediatric intensive care unit (PICU). His symptoms started 4 days before the admission with vomiting and poor feeding, for which a primary care physician prescribed antibiotics as outpatient therapy. After intubation and resuscitation, physical examination revealed a pulse rate of 154 beats per minute, respiratory rate of 70 per minute, temporal temperature of 36.5 °C, nondetectable blood pressure, and oxygen saturation of 96%. His body weight was 3700 g. He had a tense 1.5 × 1.5 cm^2^ fontanel, reactive normal-sized pupils that subsequently became miotic and nonreactive, and subcostal and suprasternal retraction. Heart sounds and abdominal examination were normal. There were multiple bolus eruptions all over the body but not in mucosal membranes (Fig. 1). Laboratory investigations showed white blood cell count of 41,000/mm^3^ with 68% neutrophils and 26% lymphocytes, hemoglobin of 10.8 g/dL, platelet count of 856,000/mm^2^, negative C-reactive protein (CRP), blood sugar of 514 mg/dL, urea of 129 mg/dL, serum creatinine (seCr) of 1.7 mg/dL, sodium (Na) of 98 mg/dL, Na (corrected for hyperglycemia) of 105 mg/dL, potassium (K) of 5.5 mg/dL, serum interleukin (IL)6 of 83.4 pg/mL, serum procalcitonin of more than 75 ng/mL, and venous blood gas (VBG) results of pH 6.80, pCO_2_ 26.2 mmHg, HCO_3:_ 4.1 meq/L, and calculated serum osmolality of 223 mOsm/kg.

On admission, he was anuric, so he receive three normal-saline stat doses (20 mg/kg). The first urine sample we could obtain was after rehydration of the patient and showed specific gravity of 1.015, pH 5, blood 3+, protein 1+, glucose 1+, WBC: 5–6/HPF, RBC: many/HPF, nitrite: negative, and bacteria: few. The patient had metabolic and respiratory acidosis, which was corrected after hydration. Table 1 presents the temporal patterns of the patient’s laboratory values.

**Table 1:** Temporal presentation of patient’s laboratory values

Time from admission	0 h	4 h	8 h	12 h	18 h	24 h	30 h	36 h	42 h
White blood cells/mm^3^	41,000	6000		24,000					
Neutrophils, %	68			48					
Lymphocytes, %	26			17					
Hemoglobin, mg/dL	10.8	11.9		10.1					
Platelets, /mm^3^	856,000	480,000		497,000					
Blood sugar, mg/dL	343	514							
Sodium (Na), mg/dL	115	98	102	113	122	131	134	135	139
Potassium (K), mg/dL	4.4	5.5	3.8	3.7	3.9	4.3			
Calcium (Ca), mg/dL	9.7	6.7	5.6						
Urea, mg/dL	110	129	108	88					
Creatinine, mg/dL		1.5	0.8	0.8					
VBG									
pH	7.22	6.9	7.33	7.40		7.37		7.37	
pCO_2_, mmHg	15.5	39.9	35	31.5		42		34	
HCO_3_^−^, meq/L	6.4	7.9	17.9	19.1		23.8		19.5	
Base excess		− 24	− 6	− 3		− 9		− 4.3	
Urinalysis									
Specific gravity				1.010					
pH				6					
White blood cells/HPF				2–3					
Red blood cells/HPF				3–4					
Bacteria/HPF				Negative					
Casts				Negative					

*VBG* venous blood gas, *HPF* high-power field

**Fig. 2:**
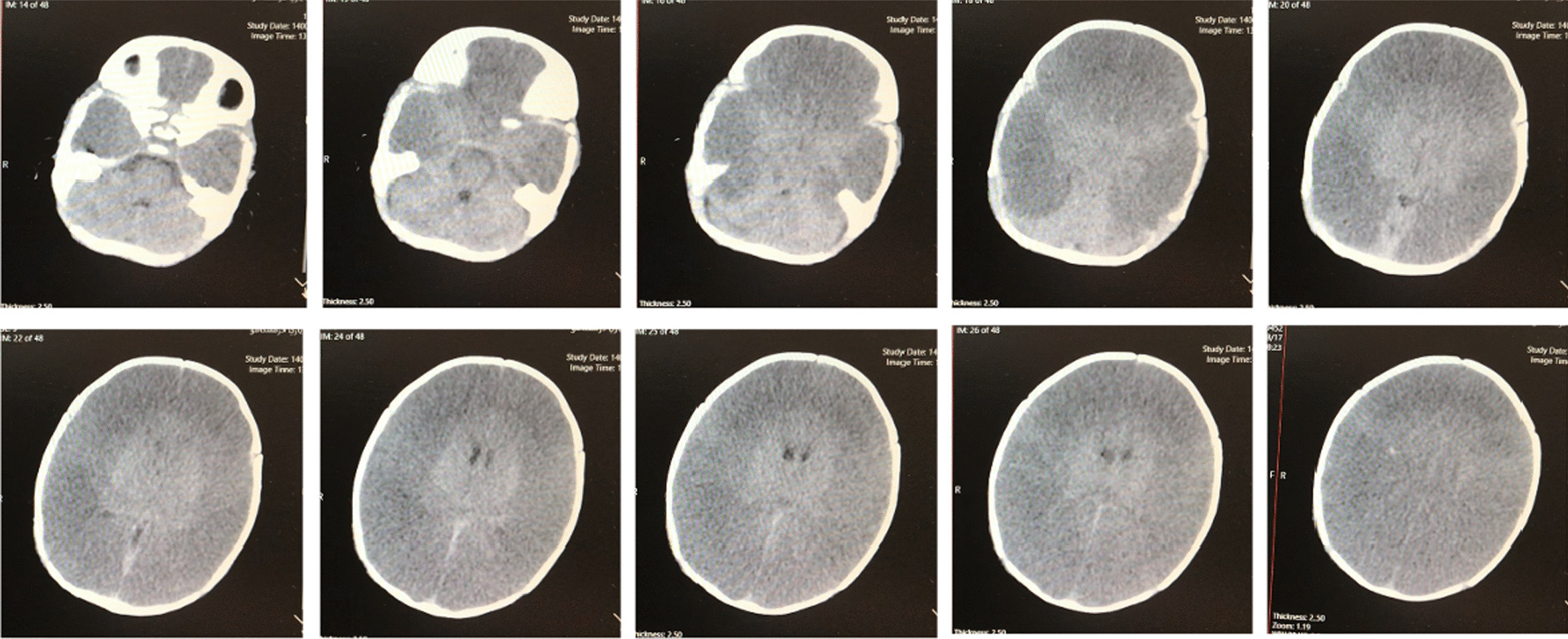
Brain computed tomography scan indicating evidence of severe diffuse cerebral edema, including brain ventricle effacement and fading of the brain sulci

He had loss of consciousness, tense fontanel, and miotic pupils. Thus, brain computed tomography (CT) scan was requested, indicating loss of differentiation between gray and white matter and effacement of cortical sulci (Fig. 2). These findings suggest severe cytotoxic brain edema. Given the evidence of severe brain edema due to hyponatremia, 3% hypertonic saline was started, and plasma sodium was corrected. Although we had attempted to correct his sodium level, because of the increasing plasma osmolality due to using mannitol and the state of hyperglycemia, this correction was not completed. The brain edema also persisted after sodium correction, but its acute symptoms disappeared. At this point, he received several doses of mannitol.

Because of the leukocytosis, he was put on intravenous cefotaxime and ampicillin. Also, a sepsis workup was done, which came back negative (negative blood culture, negative urine culture, and normal chest X-ray). However, starting antibiotics before sending culture samples could have confounded the culture results.

Subsequently, after 3 days in PICU, the symptoms of brain edema resolved, and after 4 days, he was weaned from the ventilator and extubated. Afterward, he was discharged from the PICU and transferred to the ward for further evaluation.

## Discussion

Epidermolysis bullosa is a spectrum of connective tissue disorders that cause tissue fragility, especially at skin, and may present with the involvement of many other organs such as kidneys, colon, heart, etc. [1, 13]. The principal pathological mechanism seen in EB is cell connection disorder [14] that causes tissue fragility, tissue integration impairment, and high skin permeability, so pathogen entrance, sepsis, organ involvement, water, and electrolyte imbalances are common in EB [1, 3, 9, 15].

Due to impaired skin integrity and high insensible water loss, electrolyte imbalance is a common outcome in EB patients [15]. Logically, we expected the patient to show hypernatremia and concentrated extracellular fluid (ECF). However, we report the case of an infant patient with EB with hyponatremia. In the case of EB associated with electrolyte imbalance, the disease is usually junctional, although an instance of EB simplex with electrolytes imbalance has been described [15]. In this case, we faced an ill and toxic hypovolemic patient with severe hyponatremia (Na of 98 mg/dL). According to his laboratory test results, we found high leukocyte levels, which could have indicated an infectious or inflammatory process. Some studies have mentioned that sepsis may cause hypernatremia with unknown mechanisms [16]. In this patient, sepsis could have been the cause of leukocytosis, but other findings did not match this condition, including normal CRP levels and negative blood and urine cultures, but it seems we could not rely on the laboratory results due to starting antibiotic therapy before sending culture samples.

We encountered a high level of inflammatory markers such as IL6 in the laboratory test results. This finding could indicate central nervous system (CNS) inflammation especially, in conjugation with CNS symptoms such as miotic pupils with anisocoria. Brain CT scan revealed severe cerebral edema, which continued after sodium correction.

To detect the causes of hyponatremia in this patient, lipid and protein levels in serum were evaluated and found to lie within normal limits. Therefore, pseudohyponatremia was ruled out. After initiating treatment, seCr started to decrease (from 1.7 to 0.8 mg/dL) alongside his urea, suggesting normalizing kidney function. As the patient was dehydrated, we considered hypovolemic hyponatremia. The normal findings for kidney function and normal urine sodium levels guided us toward extrarenal sodium losses in this patient, especially skin losses [16]. We attempted to correct his sodium level, but due to the increasing endogenous osmoles such as glucose (hyperglycemia) or exogenous sources such as mannitol, that shifted water out of cells and expanded the extracellular volume, this correction was not completed. Mannitol is a hypertonic solution that can act as an osmotic diuretic, causing increased urinary loss of water and electrolyte, leading to elevated urinary volume and hyponatremia. However, after treatment, the acute symptoms of our patient, such as central nervous system symptoms of bulge fontanel, miotic pupils, and respiratory distress disappeared. Furthermore, the previous scar was removed after the second brain CT scan.

## Conclusion

Many studies have confirmed that EB patients may suffer from electrolyte imbalance; however, hyponatremia is a rare condition that may also occur in these patients. This study illustrates the possibility of severe hyponatremia in patients with EB to clinicians. Appropriate clinical suspicion and knowledge on the existence of this condition in patients with EB could help healthcare workers and patients.

## Data Availability

Data are available upon a request to the corresponding author.
